# Optimization of the Time Window of Interest in Ovariectomized Imprinting Control Region Mice for Antiosteoporosis Research

**DOI:** 10.1155/2017/8417814

**Published:** 2017-10-08

**Authors:** Lei Song, Ya-nan Bi, Pan-yang Zhang, Xiao-mei Yuan, Ying Liu, Yue Zhang, Ju-yang Huang, Kun Zhou

**Affiliations:** ^1^Institute of Traditional Chinese Medicine, Tianjin University of Traditional Chinese Medicine, Tianjin 300193, China; ^2^Key Laboratory of Formula of Traditional Chinese Medicine, Tianjin University of Traditional Chinese Medicine, Ministry of Education, Tianjin 300193, China; ^3^Tianjin State Key Laboratory of Modern Chinese Medicine, Tianjin 300193, China; ^4^College of Veterinary Medicine, Inner Mongolia Agricultural University, Hohhot 010018, China

## Abstract

This study was performed to determine the optimal window of time during which the properties of osteoporosis are obvious and to explore the best region of interest for microstructural evaluation in antiosteoporosis research in an ovariectomized mouse model by examining changes in micro-computed tomography parameters and serum indices. Ovariectomized mice and sham-operated mice were randomly divided into five groups. At the end of the 4th, 8th, 12th, 16th, and 20th weeks after ovariectomy, the microstructure of the proximal tibia and distal femur was scanned by micro-computed tomography and blood samples were collected to detect serum biochemical indicators including alkaline phosphatase, osteocalcin, N-terminal propeptide of type I procollagen (P1NP), and C-terminal telopeptide fragment of type I collagen (CTX1). The trabecular number and connectivity density decreased while the trabecular thickness and trabecular separation increased, indicating substantial changes in the trabecular microstructure of both the tibia and femur and significant changes in bone turnover after ovariectomy, as indicated by lower levels of serum alkaline phosphatase, osteocalcin, and P1NP and higher level of CTX1 in the ovariectomy than sham group. The proximal tibia from weeks 8 to 16 after ovariectomy was optimal for osteoporosis research in this model.

## 1. Introduction

Osteoporosis is a systemic skeletal disease characterized by decreases in the mass and density of bone, ultimately resulting in bone fragility and a higher risk of fracture [1]. The most severe complication of osteoporosis is bone fracture, which is associated with a high risk of disability and mortality [2, 3]. According to one study, about 50% of women and 20% of men above the age of 50 years will develop an osteoporosis-related fracture as the prevalence of osteoporosis increases [4]. By 2050, the worldwide incidence of hip fracture is expected to reach 6.26 million in comparison with only 1.66 million in 1990 [5]. The increasing prevalence of osteoporosis will not only result in risks for elderly people but may also present socioeconomic burdens [6]. Approximately $19 billion was spent to treat osteoporotic-related fractures in 2005 in the US, and this is expected to triple by the year 2040 [5]. Thus, the research and management of osteoporosis are a socioeconomic priority.

Hormonal deficiency is reportedly the main cause of osteoporosis, especially in postmenopausal and ovariectomized (OVX) women [7]. Deficiency of estrogen directly affects bone turnover by stimulating osteoclast activity and inhibiting osteoblast activity [8, 9]. Female OVX rats, which mimic the postmenopausal hormonal changes that occur in humans [10], are currently the most widely used animal models to evaluate changes in the trabecular bone architecture, serum indices after ovariectomy, and treatment results of antiosteoporotic drugs [11, 12]. However, although mouse models require less money and medical resources, especially drugs that are difficult to purify and synthesize, few systematic studies have been performed on mouse models of osteoporosis. Chen et al. [1] used mouse models of osteoporosis, but they performed a drug efficacy study rather than evaluate the different parameters of the model. Laib et al. [13] studied the trabecular bone microstructure of osteoporosis, but they used rats and only established one control group for all OVX groups at different time points. Moreover, only femurs were scanned by micro-computed tomography (micro-CT) to analyze the association between the bone micro architecture and osteoporosis.

We assessed the micro-CT parameters and serum indices in an OVX mouse model to investigate the optimal window of time during which the properties of osteoporosis are obvious and determine which body parts are most effective for microstructural examination of osteoporosis.

## 2. Materials and Methods

### 2.1. Ethics Statement

All animal studies were performed strictly with the approval of the Laboratory Animal Ethics Committee of Tianjin University of Traditional Chinese Medicine (permit number: TCM-LAEC 2015015). All experiments and procedures were conducted under anesthesia, and all attempts were made to minimize suffering.

### 2.2. Animals and Study Design

All animal experiments involved normal mice. Female imprinting control region mice weighing 20 g were obtained from Beijing HFK Bioscience Technology Co., Ltd. (Beijing, China). They were acclimatized for 30 days before the experiment. All mice were housed in plastic cages with ventilated tops and stainless steel lips under controlled conditions of light (12 h light/dark illumination cycles), temperature (22°C ± 2°C), and humidity (65% ± 5%).

In the experiment, the 8-week-old mice underwent ovariectomy to induce female osteoporosis, and equal numbers of mice underwent a sham operation after adaptation. All mice in the OVX group underwent bilateral ovariectomy, and those in the sham surgery group underwent the same procedure except that the ovaries were identified and then preserved. The mice were maintained in a warm environment and checked every 15 minutes until they fully recovered. The surviving OVX mice were randomly divided into five groups for measurements at five different time points; the mice in the sham group were also divided into five groups as controls. Each group had 10 mice.

The mice in each of the five OVX groups were killed at postoperative weeks 4, 8, 12, 16, and 20, respectively. The mice in each of the sham groups (control groups) were also killed at the same time points to match the OVX groups.

During all studies, the mice in the OVX groups were pair-fed with those in the sham groups to prevent postovariectomy hyperphagia.

### 2.3. Micro-CT Analyses

The morphological parameters of trabecular bone were qualitatively and quantitatively analyzed using a micro-CT system (vivaCT 40; Scanco Medical AG, Brüttisellen, Switzerland).

In all groups, the right femur and tibia were excised and fixed in 10% phosphate-buffered formalin. Before scanning, the bones were transferred to 70% ethanol, and the specimens were then dried in air for 15 to 20 min. The right distal femur and proximal tibia of mice from the sham groups and OVX groups were scanned to evaluate the microstructure of the distal femur and proximal tibia. The distal femur was scanned from the growth plate distally in 5 slices, and 80 serial slices were chosen for the evaluation; the length was about 0.84 mm. The proximal tibia was scanned using the same procedure except that the scan was begun from the growth plate proximally in five slices. A reconstruction of the bitmap data set was obtained and employed to build the three-dimensional (3D) model, and structural analyses were performed using Scanco Medical version 6.0 software.

The following parameters were calculated for quantitative analysis using direct results (without the plate model): total volume (TV), bone volume (BV), density of bone (of BV), density of tissue (of TV), relative bone volume (BV/TV), trabecular thickness (Tb.Th), trabecular separation (Tb.Sp), trabecular number (Tb.N), structure model index (SMI), and connectivity density (Conn.D).

### 2.4. Biochemical Analyses of Serum Samples

Blood samples were collected from the mice, and the serum was centrifuged at 3500 rpm at 4°C for 20 min. The separated sera were analyzed for detection of biochemical markers, including C-terminal telopeptide fragment of type I collagen (CTX1), N-terminal propeptide of type I procollagen (P1NP), osteocalcin (OC), and alkaline phosphatase (ALP) [14]. CTX1 was measured by a specific enzyme-linked immunosorbent assay (ELISA) kit (CEA665Mu; Cloud-Clone Corp., Katy, TX, USA). P1NP was measured by an ELISA kit for mouse P1NP (SEA957Mu; Cloud-Clone Corp.) according to the manufacturer's instructions. OC was analyzed using a standard commercial ELISA kit for OC (SEA471Mu; Cloud-Clone Corp.). ALP was determined using an autoanalyzer (7020; Hitachi, Tokyo, Japan).

### 2.5. Statistical Analysis

Data regarding bone microstructure and serum biomarkers were analyzed using an analysis of variance (ANOVA) and *t*-test to determine whether there were significant differences between the sham and OVX groups. All numerical results are shown as mean ± standard error of the mean. SPSS software was utilized for all statistical analyses. Statistical significance was defined as a* p *value of <0.05.

## 3. Results

### 3.1. Animal Weight Changes

**Figure 1:**
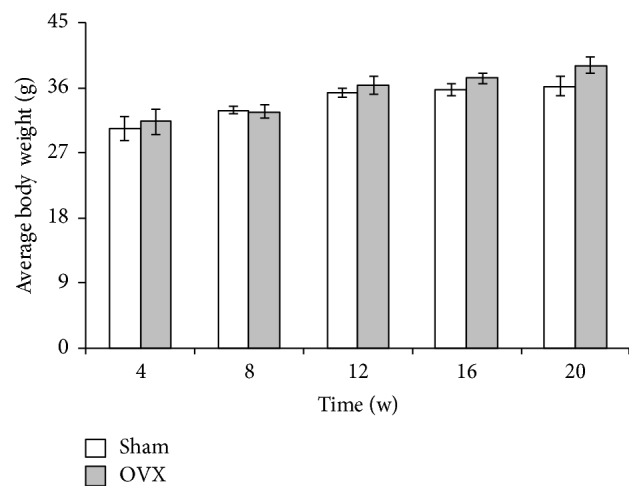
*Average body weight of mice during 20-week study period*. Sham, sham-operated group (control group); OVX, ovariectomized group (experimental group). The OVX and sham groups were compared at each time point, but no significant differences were observed.

At the end of every measurement time point, the average weight of the mice from the sham and OVX groups was statistically analyzed. Their average body weight slightly increased, but no significant differences were observed (*p* > 0.05) (Figure 1).

### 3.2. Structural Parameters in the Tibia

**Figure 2:**
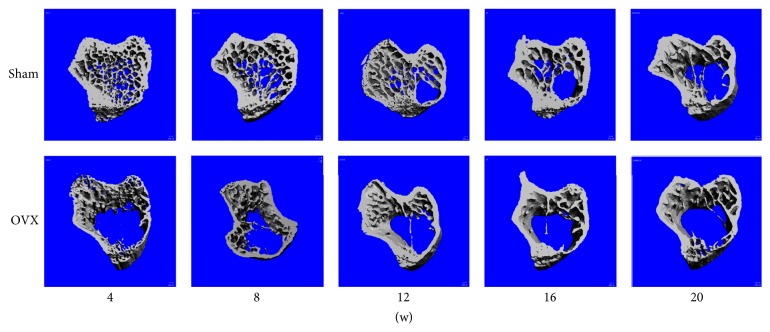
*Tibial 3D images at different time points*. Tibial 3D images in the sham and OVX groups were built and are presented in pairs at different time points.

The 3D trabecular bone microstructure of the proximal tibia in the sham and OVX groups at different time points was analyzed by 3D micro-CT and is shown in Figure 2. More loss of the trabecular microstructure was observed in the OVX than sham group. The OVX group showed more evident degeneration of the microstructure from weeks 8 to 16 after the operation.

**Figure 3:**
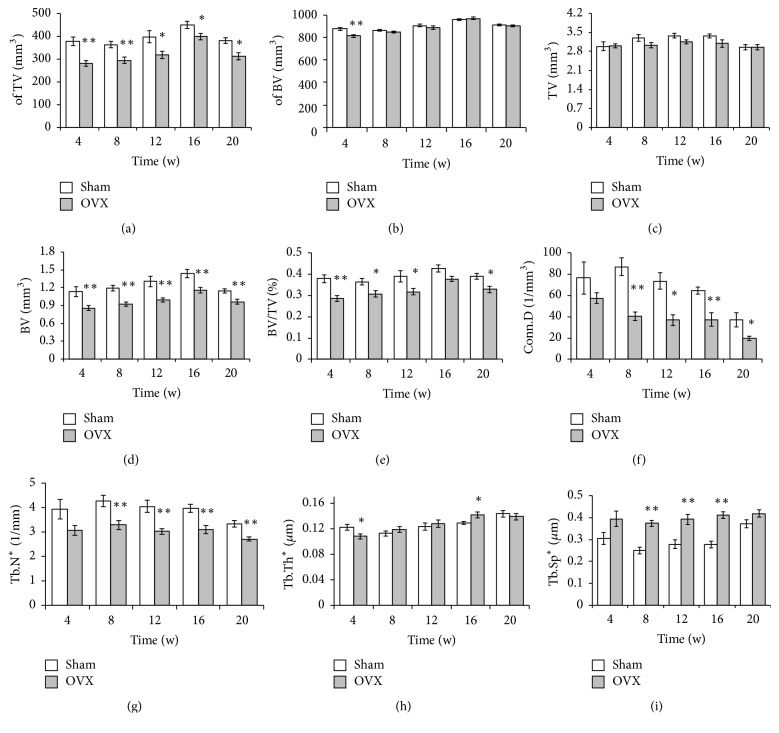
*Structural parameters of the tibia*. The structural parameters of the trabecular bone of the tibia were measured with micro-CT. Sham, sham-operated group (control group); OVX, ovariectomized group (experimental group). The OVX and sham groups were compared at each time point. ^*∗*^*p* < 0.05; ^*∗∗*^*p* < 0.01 versus sham. Tb.N^*∗*^, Tb.Th^*∗*^, and Tb.Sp^*∗*^ indicate that the algorithm was recommended by the software.

Results of the analysis of quantitative parameters of trabecular microstructural changes are shown in Figure 3. The results of sham groups by ANOVA showed that parameters of trabecular microstructure, except BV/TV and Tb.N (*p* > 0.1), had significant difference (*p* < 0.05) at different time points, which indicated the control group was unstable at 5 different time points, so *t*-test was adopted to determine whether there were significant differences between the OVX group and the sham group. Deterioration of the trabecula was notable in the comparison of the sham and OVX groups. At different time points, the OVX group showed significant reductions in the TV (Figure 3(a)), BV (Figure 3(d)), BV/TV (Figure 3(e)), Conn.D (Figure 3(f)), and Tb.N (Figure 3(g)) and elevation in the Tb.Sp (Figure 3(i)). Significant differences in Tb.Th were also observed. As shown in Figure 3(h), Tb.Th was significantly lower at week 4 in the OVX group but prominently exceeded that in the sham group at week 16 in the tibia (Figure 3(h)). Although the TV and BV were altered, they showed no significant differences (Figures 3(b) and 3(c)).

**Figure 4:**
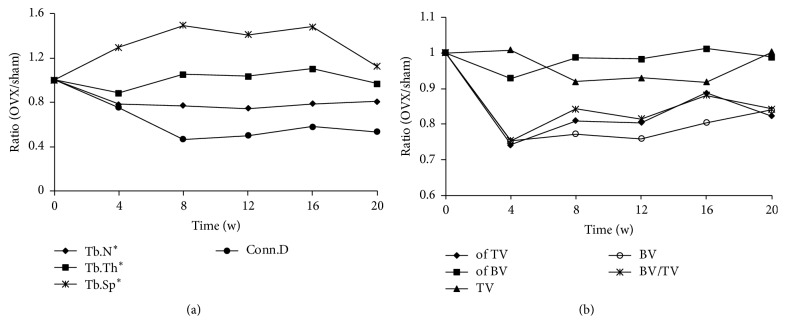
*Changes in the tibial microstructure*. The ratios (OVX/sham) of bone parameters measured by micro-CT were analyzed to observe the relative changes in each parameter. Tb.N^*∗*^, Tb.Th^*∗*^, and Tb.Sp^*∗*^ mean that the algorithm was recommended by the software.

Taking the unstable factors of the control group into consideration, the ratios (OVX/sham) of the bone parameters detected by micro-CT were assessed to observe the trends in variation (Figure 4). Tb.N and Conn.D showed an abrupt decrease from the beginning of the study to week 8, after which the levels remained stable with small fluctuations. Tb.Th dropped in the first 4 weeks, increased gradually from weeks 4 to 8, and then exhibited small increases and decreases. Conversely, Tb.Sp increased during the first 8 weeks postoperatively but unexpectedly decreased during the last 4 weeks (Figure 4(a)).

BV/TV, BV, of BV, and of TV showed similar trends in that they dropped sharply in the first 4 weeks and then slightly increased. The TV curves fluctuated within a minimal range (Figure 4(b)).

### 3.3. Structural Parameters of the Femur

**Figure 5:**
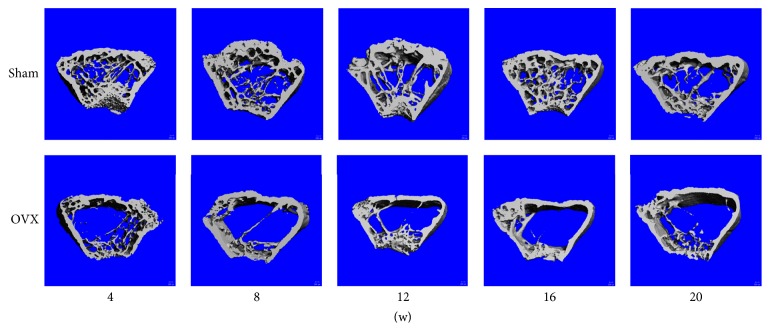
*Femoral 3D images at different time points*. Femoral 3D images in the sham and OVX groups were built and are presented in pairs at different time points.

Femoral 3D images in the sham and OVX groups are shown in Figure 5. A compact trabecular network was observed in the sham mice, and the OVX group exhibited more evident degeneration of the microstructure from weeks 8 to 16 postoperatively.

**Figure 6:**
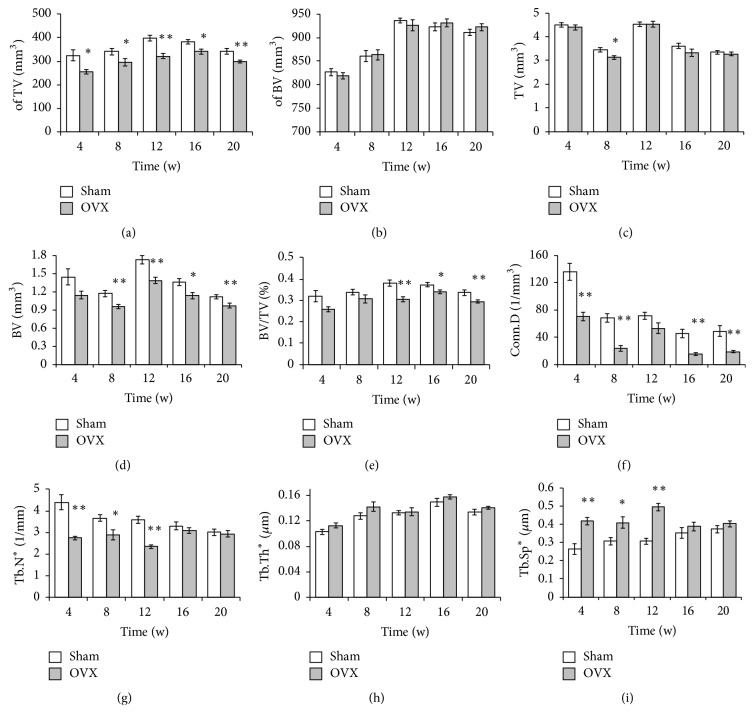
*Structural parameters of the femur*. The structural parameters of the trabecular bone of the femur were measured with micro-CT. Sham, sham-operated group (control group); OVX, ovariectomized group (experimental group). The OVX and sham groups were compared at each time point. ^*∗*^*p* < 0.05; ^*∗∗*^*p* < 0.01 versus sham. Tb.N^*∗*^, Tb.Th^*∗*^, and Tb.Sp^*∗*^ mean that the algorithm was recommended by the software.

The parameters of the distal femur were also analyzed and are exhibited in Figure 6. The results of sham groups by ANOVA showed that parameters of trabecular microstructure all had significant difference (*p* < 0.05) at different time points. Significant differences in of TV by *t*-test were detected throughout the study, and the levels were lower in the OVX than sham group in the distal femur (Figure 6(a)). In contrast with BV from tibia, BV of femur showed no significant difference at week 4 (Figure 6(d)). The BV/TV of the femur was also lower in the OVX group, showing significant differences except at weeks 4 and 8 (Figure 6(e)). Significant differences in Conn.D between the OVX and sham groups were observed at all time points except week 12 (Figure 6(f)). Tb.N of the femur in the OVX group was decreased but showed no significant differences at weeks 16 and 20 (Figure 6(g)). Tb.Sp in the OVX group was substantially higher than that in the sham group, but only at the first three time points (Figure 6(i)). In the femur, of BV, TV, and Tb.Th changed slightly but showed no significant differences (Figures 6(b), 6(c), and 6(h)). The general trends approximated those of the corresponding parameters in the proximal tibia.

**Figure 7:**
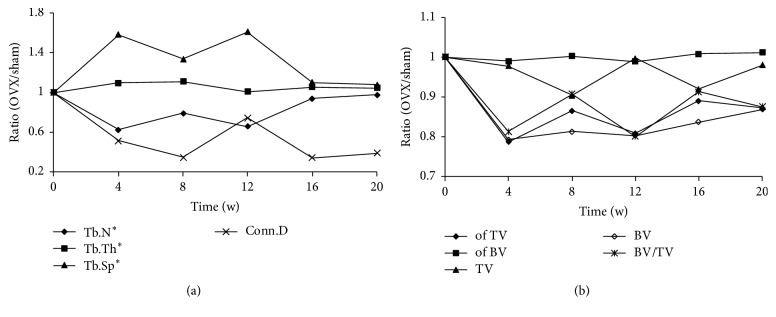
*Changes in the microstructure of the femur*. The ratios (OVX/sham) of bone parameters measured by micro-CT were analyzed to observe the relative changes in each parameter. Tb.N^*∗*^, Tb.Th^*∗*^, and Tb.Sp^*∗*^ mean that the algorithm was recommended by the software.

The parameters of the distal femur were assessed using the same means applied in the proximal tibia; however, the ratio (OVX/sham) of different points in the whole experiment fluctuated strongly. Tb.Sp increased during the first 12 weeks and decreased to normal during the last 8 weeks. Tb.Th was relatively stable throughout the experiment, remaining at a normal level. Tb.N and Conn.D, as in the tibia, decreased rapidly in the first 4 weeks and then recovered with time (Figure 7(a)).

### 3.4. Biochemical Analyses of Serum Samples

**Figure 8:**
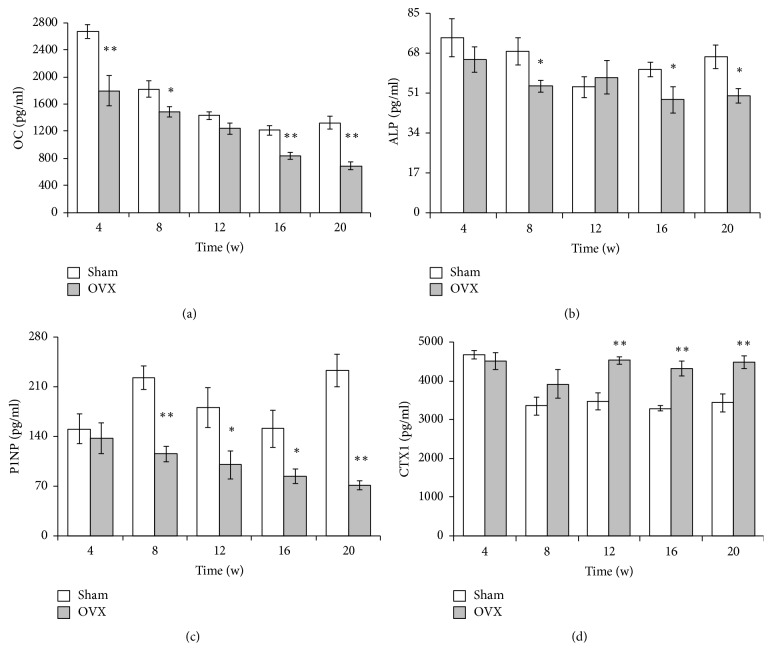
*Serum levels of OC, ALP, P1NP, and CTX1*. Sham, sham-operated group (control group); OVX, ovariectomized group (experimental group). The OVX and sham groups were compared at each time point. ^*∗*^*p* < 0.05; ^*∗∗*^*p* < 0.01 versus sham.

**Figure 9:**
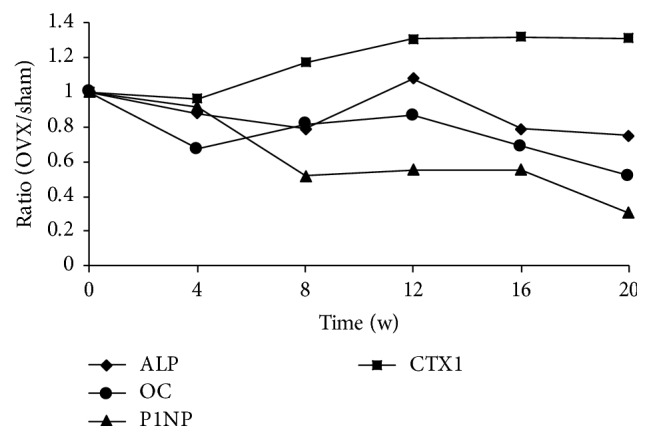
*Ratios (OVX/sham) of OC, ALP, P1NP, and CTX1*. To observe the relative change of the parameters, the ratio (OVX/sham) derived from bone turnover markers was presented.

The serum levels of OC, ALP, and P1NP as bone formation markers and CTX1 as a bone resorption marker are summarized in Figure 8. Similarly, the results of sham groups by ANOVA showed that OC, ALP, P1NP, and CTX1 had significant difference (*p* < 0.05) at different time points, so *t*-test was adopted to determine whether there were significant differences between the OVX group and the sham group. Comparison between the OVX and sham groups at different time points showed obvious reductions in the bone formation markers OC, ALP, and P1NP in the OVX group; however, ALP in the sham group at week 12 was higher than that in the OVX group (Figures 8(a)–8(c)). The CTX1 level was higher in the OVX than sham group from week 12, indicating increased bone resorption activity (Figure 8(d)). Additionally, given that the animals might have been affected by various factors, the ratios (OVX/sham) were analyzed and graphed (Figure 9). The trend revealed that CTX1 increased with time and that OC, ALP, and P1NP decreased with time.

## 4. Discussion

Prevention and treatment of osteoporosis are considered important because osteoporosis typically leads to a higher probability of fractures, which increase patient disability and the economic burden on the health care system [15]. We investigated changes in the microstructure of bone and various serum markers with time to identify the optimal window of time and most appropriate body parts for assessment of osteoporosis.

In this study, the OVX and sham groups showed no differences in body weight at various time points. Caloric intake and food consumption may account for this result. The three diets provided to the mice were equicaloric, and food consumption was equally matched in all groups. Our result is in agreement with a previous study of rats [16, 17].

The lumbar spine and proximal femur are common sites for dual-energy X-ray absorptiometry examination, the most widely adopted diagnostic method for osteoporosis [18]. Osteoporosis is commonly thought to mainly involve the trabecular bone; however, the percentage of trabecular bone in the vertebrae is much lower than generally thought [19]. Ryu et al. [20] reported that changes in the bone mineral density of the lumbar spine were slower to occur than those in the more commonly examined bones, such as the femoral bone. In our examination of the proximal femur, we scanned the femoral neck of mice, which has very few trabeculae that are not obvious. Taking factors such as time, cost, radiation exposure, and the observation index into consideration, we choose the distal femur and proximal tibia, both very commonly used parts in daily activities, as the research object. Ultimately, we scanned the distal femur and proximal tibia of the mice and analyzed their microstructure.

Disruption of the bone microstructure, such as that evidenced by a reduction in Tb.N and Conn.D and an increase in Tb.Sp and Tb.Th [21], is the hallmark of osteoporosis [22]. Analogical results regarding the changes in the trabecular microstructure have been reported in rats [23, 24], but few such findings have been reported in mice. In the current study, these characteristics of osteoporosis were observed at different time points. We found that the microstructure of the proximal tibia changed markedly, including reductions in TV, BV, BV/TV, Tb.N, and Conn.D and increases in Tb.Sp. In the distal femur, the changes between the sham and OVX groups were similar to those in the proximal tibia, which showed great changes in the trabecular microstructure. The Conn.D of the bony trabeculae is the basic characteristic of the 3D network that maintains the strength of the skeleton, and BV and Conn.D are reportedly associated with bone loss [13]. In the present study, both the BV and Conn.D of the proximal tibia and distal femur in the OVX group declined substantially, implying obvious bone loss. Moreover, significant differences in the TV, BV, and BV/TV were observed in both the proximal tibia and distal femur from weeks 8 to 16, confirming changes in the microstructure in the OVX group.

We also observed a diminished trabecular microstructure after ovariectomy, and the findings in the proximal tibia showed an apparent and stable change compared with the distal femur. Tb.Sp of the proximal tibia increased while the Tb.Sp and Conn.D decreased. Interestingly, however, Tb.Sp was low at week 20. We speculated that time, in addition to large individual differences, was already not the main influence factor that determined Tb.Sp at week 20 and later. The levels were basically stable from weeks 8 to 16 after ovariectomy in both the proximal tibia and distal femur; however, the curves of the distal femur fluctuated acutely.

We further evaluated the serum indicators at each time point to investigate the changes in bone turnover markers. For sham groups, our results showed that there are the substantial variations in OC, ALP, and P1NP results. These variations may be related to many factors, such as day-night rhythm, age, and individual difference. Few researches reported the temporal changes in mice, and the serum OC had sustained decrease trend with age in our study; we think that the change should be related to the age. As others, now we are not sure what exactly the cause is. However, this may be the strong support to our design of one control group at each point. If there is only one control group at the beginning, we will get different results and the results may be incorrect at some time points. Compared with the sham group at each point, the results revealed significant changes in bone turnover after ovariectomy as indicated by the lower levels of serum ALP, OC, and P1NP and higher level of CTX1. Bone formation and bone resorption jointly determine the level of bone metabolism. Markers of bone formation and bone resorption are a means of predicting bone metabolism [25]. Serum indicators produced by osteoblasts, such as ALP, P1NP, and OC, can reflect osteoblast functions and bone formation activities [26]. Our results revealed lower OC and P1NP levels in the OVX than sham group. The ALP level in the OVX group was also lower than that in the sham group except at week 12, at which point it was unexpectedly higher. We speculate that large individual differences in the experimental animals, such as body weight, exercise level, and/or malabsorption [27], may be responsible for this phenomenon. The gradual decline in the ALP, P1NP, and OC in the OVX group indicated that osteoblast activity and bone formation were weakened. CTX1, a predictor of osteoclast activity, is the most extensively studied and used parameter for assessment of bone resorption [28]. In the present study, its level was higher in the sham than OVX group and showed significant differences at weeks 12, 16, and 20, indicating intensive osteoclast activity. Additionally, P1NP and CTX1 are recommended as the reference bone formation and resorption markers, respectively, by the International Osteoporosis Foundation and International Federation of Clinical Chemistry [27]. After surgery, the CTX1 level increased and the P1NP level decreased, indicating that bone resorption exceeded bone formation. Due to a combination of factors, the serum indices remained relatively stable for 8 to 16 weeks postoperatively.

Our* in vivo* experiment clearly proves that ovariectomy in mice results in apparent changes in bone turnover markers and the trabecular bone structure of the distal femur and proximal tibia. Given the trends in all parameters, the proximal tibia was the most effective body part for assessment of osteoporosis in our mouse model, and 8 to 16 weeks was the optimal window of time. However, only distal femur and proximal tibia of ICR mice were analyzed in the experiment; more specific sites, such as hip, spine, femoral neck, and other sites, should be explored. Besides, the results of morphological parameters of trabecular bone, bone turnover markers, and appropriate time window may have some differences in experimental animals of different strains, for example, ICR mice and C57 mice. But we believe the difference will not affect the value of this article.

## 5. Conclusion

In summary, 8 weeks is adequate for avoiding long waiting periods in the experimental setting and ensuring a large enough significant difference in the main indices, which include morphological parameters of trabecular bone (e.g., Conn.D, Tb.N, and Tb.Sp) and bone turnover markers (e.g., OC, ALP, P1NP, and CTX1). For 8 to 16 weeks after ovariectomy, the mouse models presented remarkable changes in trabecular morphological parameters and bone turnover markers of osteoporosis and are thus suitable for further research on disease mechanisms and pharmacotherapy.
